# Prevention of Mental Disorders in Children of Parents with Psychiatric Illness

**DOI:** 10.1192/j.eurpsy.2026.10997

**Published:** 2026-07-31

**Authors:** O. Amiot, E. Petiau, D. Waintrater, L. Koziatek Molina, I. Fradi, M. C. Beaucousin

**Affiliations:** 93, Etablissement Public de Santé Ville Evrard, Saint Ouen, France

## Abstract

**Introduction:**

Parental mental illness is a major public health issue due to its frequency and its impact on children’s development. When parents experience psychiatric instability, their ability to meet children’s emotional and educational needs may be compromised, leading to family dysfunction, lack of emotional support, and increased exposure to stress. As a result, children of parents with mental illness are at higher risk of emotional, academic, and social difficulties. Despite this, parental roles and children’s experiences remain neglected within adult psychiatric care.

Framed systemically, illness is a family event: many children act as young carers and experience role reversal, isolation, and stigma.

Evidence from Kidstime, a preventive, multifamily, non-therapeutic model, shows benefits from age-appropriate explanations, peer contact, and a reliable adult, supporting our local implementation.

**Objectives:**

Implement a multifamily group to:

1. break silence and reduce stigma around parental mental illness;
2. provide children with clear, age-appropriate information and a supportive space;
3. strengthen parents’ confidence and competence;
4. reduce isolation by fostering peer support and links with care teams/community resources;
5. promote prevention at two levels: relapse prevention in parents and mental-health promotion in children.

**Methods:**

UMPVE is an eight-session, monthly multifamily psychoeducational program for adults in psychiatric care, their partners, and children aged 5–18. Sessions follow a fixed structure (parallel parent/child groups, creative activities, shared discussion, informal time). Families are referred by their psychiatrist and attend a pre-admission interview; workshops are co-facilitated by a multidisciplinary team (psychiatrists, psychologists, drama facilitator) in a safe, non-stigmatizing setting. A pilot started in December 2024.

Evaluation includes sociodemographic and clinical data, attendance, and qualitative feedback from parents, children, and professionals.

**Results:**

Preliminary observations indicate strong feasibility and acceptability. Parents reported reduced isolation, greater support, and increased confidence in their parental role. Children described the workshops as safe spaces to understand their parent’s illness and express emotions without guilt or fear. Professionals noted improved family communication and a strengthened therapeutic alliance.

These first experiences align with international findings on the benefits of multifamily preventive groups.

**Conclusions:**

UMPVE shows that family-centered, multifamily interventions are feasible and well accepted in adult psychiatry. By addressing children’s needs, supporting parents, and reducing isolation, this program responds to a critical but often overlooked issue. Our early experience suggests that such groups may help break the cycle of intergenerational transmission of mental illness. Broader dissemination and further evaluation are warranted.

**Disclosure of Interest:**

None Declared

